# Long-term kidney outcomes associated with telitacicept in patients with IgA nephropathy: a 24-month retrospective comparative cohort study

**DOI:** 10.1093/ckj/sfag309

**Published:** 2026-09-02

**Authors:** Cheng Hanbing, Liu Youxia, Li Hongfen, Wang Fanghao, Xing Yue, Li Wenying, Wu Zhanfei, Zhang Kai, Wang pengzhi, Xu Mengke, Jia Junya

**Affiliations:** Department of Nephrology, Kidney Disease Medical Center, Tianjin Medical University General Hospital, National Key Clinical Specialty, Tianjin Key Medical Discipline, Tianjin, China; Department of Nephrology, Kidney Disease Medical Center, Tianjin Medical University General Hospital, National Key Clinical Specialty, Tianjin Key Medical Discipline, Tianjin, China; Department of Nephrology, Kidney Disease Medical Center, Tianjin Medical University General Hospital, National Key Clinical Specialty, Tianjin Key Medical Discipline, Tianjin, China; Department of Nephrology, Kidney Disease Medical Center, Tianjin Medical University General Hospital, National Key Clinical Specialty, Tianjin Key Medical Discipline, Tianjin, China; Department of Nephrology, Kidney Disease Medical Center, Tianjin Medical University General Hospital, National Key Clinical Specialty, Tianjin Key Medical Discipline, Tianjin, China; Department of Nephrology, Tianjin Medical University General Hospital Airport Hospital, Tianjin, China; Department of Nephrology, Kidney Disease Medical Center, Tianjin Medical University General Hospital, National Key Clinical Specialty, Tianjin Key Medical Discipline, Tianjin, China; Department of Nephrology, Kidney Disease Medical Center, Tianjin Medical University General Hospital, National Key Clinical Specialty, Tianjin Key Medical Discipline, Tianjin, China; Department of Nephrology, Kidney Disease Medical Center, Tianjin Medical University General Hospital, National Key Clinical Specialty, Tianjin Key Medical Discipline, Tianjin, China; Department of Nephrology, Kidney Disease Medical Center, Tianjin Medical University General Hospital, National Key Clinical Specialty, Tianjin Key Medical Discipline, Tianjin, China; Department of Nephrology, Kidney Disease Medical Center, Tianjin Medical University General Hospital, National Key Clinical Specialty, Tianjin Key Medical Discipline, Tianjin, China

To the Editor,

Dual blockade of B-cell activating factor (BAFF) and a proliferation-inducing ligand (APRIL) has become an important emerging therapeutic strategy for immunoglobulin A nephropathy (IgAN), in which persistent proteinuria and declining estimated glomerular filtration rate (eGFR) remain key determinants of long-term kidney outcome [1, 2]. Recent phase 2 and interim phase 3 studies have demonstrated that telitacicept significantly reduces proteinuria in patients with IgAN [3, 4]. However, evidence regarding its long-term effectiveness on kidney function trajectories in routine clinical practice remains limited. We therefore evaluated 24-month kidney outcomes after telitacicept therapy in a single-center retrospective comparative cohort.

In total, 682 biopsy-confirmed IgAN patients were screened from June 2023 to April 2026. Following eligibility assessment and 1:2 propensity-score matching, 10 telitacicept-treated patients and 20 matched controls from the same source population were enrolled, all with complete baseline data and adequate follow-up (Fig. S1). Supplementary Table 1 shows full baseline characteristics for evaluating postmatching balance. Median baseline eGFR was 69.5 versus 61.4 mL/min/1.73 m^2^, and median 24-h urinary protein excretion was 1.45 versus 1.08 g/day in the telitacicept and control groups, respectively.

Telitacicept dosing was individualized during follow-up according to clinical response and treatment tolerance. Most patients were treated with a 160-mg-based initial regimen, followed by individualized dose reduction and/or extension of the dosing interval during follow-up. No patient permanently discontinued telitacicept before completion of the 24-month follow-up, although dose and dosing frequency were adjusted during treatment. Individual treatment trajectories are shown in Fig. S2.

Individual eGFR trajectories during telitacicept treatment are shown in Fig. 1A. During follow-up, mean eGFR progressively increased to 83.4 ± 21.9 mL/min/1.73 m^2^ at month 24, whereas the control group showed a less pronounced change in kidney function over time (Fig. 1B). Individual annualized eGFR slopes were significantly more favorable in telitacicept-treated patients compared with controls (*P* = .013), indicating a more favorable long-term renal function trajectory.

**Figure 1:**
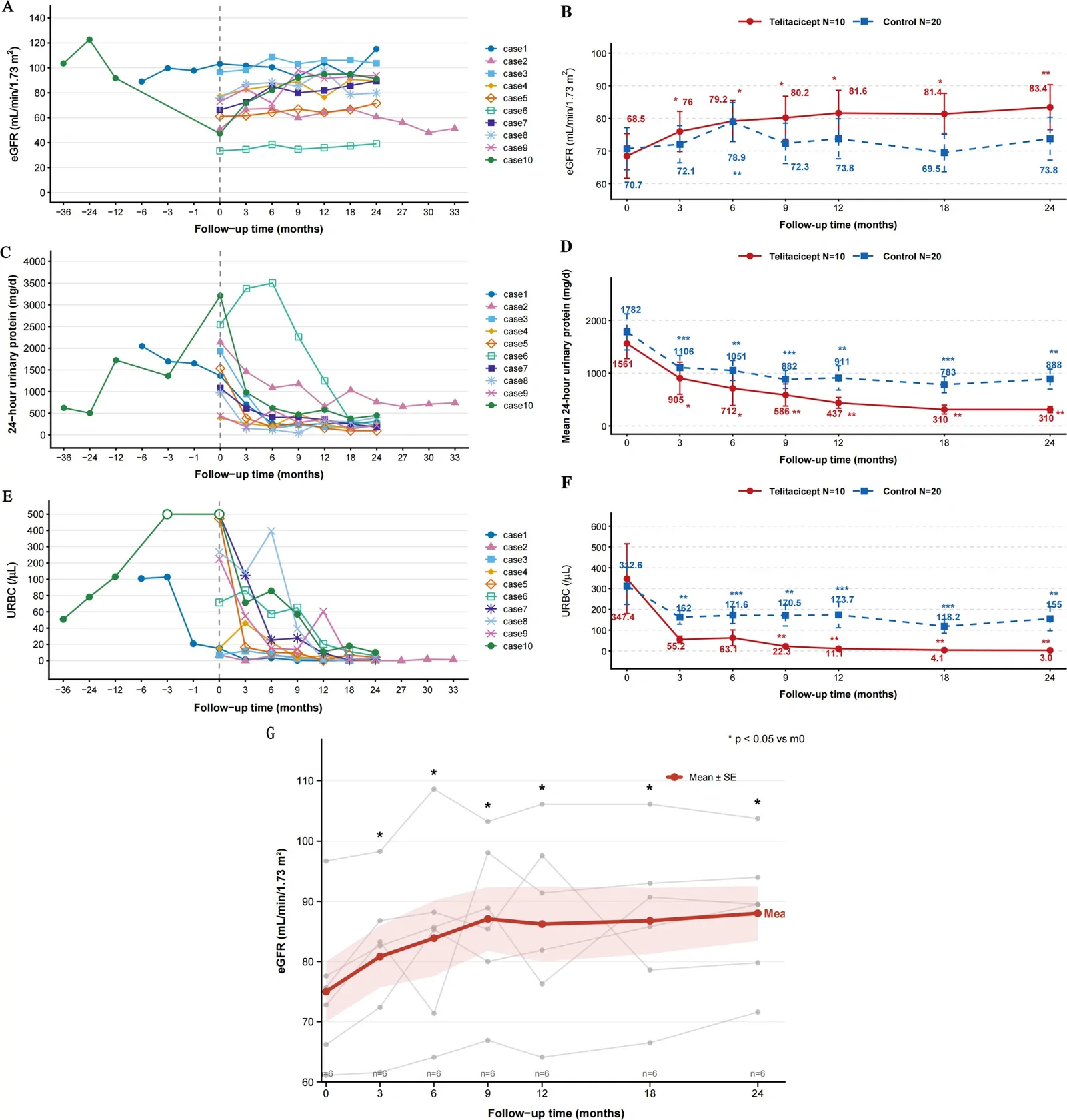
Kidney outcomes in patients with immunoglobulin A nephropathy treated with telitacicept and contemporaneous controls.(A) Individual estimated glomerular filtration rate (eGFR) trajectories before and after telitacicept initiation. (B) Mean eGFR over time in the telitacicept and control groups. (C) Individual 24-h urinary protein excretion trajectories before and after telitacicept initiation. (D) Mean 24-h urinary protein excretion over time in the telitacicept and control groups. (E) Individual urinary red blood cell (URBC) count trajectories before and after telitacicept initiation. (F) Mean URBC count over time in the telitacicept and control groups. (G) Sensitivity analysis of eGFR over time after excluding patients who received intensive glucocorticoid or immunosuppressive therapy. In panels A, C, and E, each line represents an individual patient, and the vertical dashed line indicates telitacicept initiation at month 0. In panels B, D, and F, points and error bars represent the mean ± standard error (SE), and numerical labels indicate mean values at each time point. In panel G, thin lines represent individual patient trajectories, and the bold line with shaded area represents the mean ± SE. Asterisks indicate statistically significant differences from baseline within the corresponding group: P < .05, P < .01, and P < .001.outcomes in patients with immunoglobulin A nephropathy treated with telitacicept and contemporaneous controls.(A) Individual estimated glomerular filtration rate (eGFR) trajectories before and after telitacicept initiation. (B) Mean eGFR over time in the telitacicept and control groups. (C) Individual 24-h urinary protein excretion trajectories before and after telitacicept initiation. (D) Mean 24-h urinary protein excretion over time in the telitacicept and control groups. (E) Individual urinary red blood cell (URBC) count trajectories before and after telitacicept initiation. (F) Mean URBC count over time in the telitacicept and control groups. (G) Sensitivity analysis of eGFR over time after excluding patients who received intensive glucocorticoid or immunosuppressive therapy. In panels A, C, and E, each line represents an individual patient, and the vertical dashed line indicates telitacicept initiation at month 0. In panels B, D, and F, points and error bars represent the mean ± standard error (SE), and numerical labels indicate mean values at each time point. In panel G, thin lines represent individual patient trajectories, and the bold line with shaded area represents the mean ± SE. Asterisks indicate statistically significant differences from baseline within the corresponding group: *P* < .05,**P < .01, and***P < .001

Proteinuria decreased rapidly and persistently following telitacicept initiation. Individual proteinuria changes are shown in Fig. 1C. Mean 24-h urinary protein decreased from 1.56 g/day at baseline to 0.90, 0.71, 0.59, 0.44, 0.31, and 0.31 g/day at months 3, 6, 9, 12, 18, and 24, respectively (Fig. 1D). At month 24, complete remission occurred in 6 of 10 telitacicept-treated patients versus 6 of 20 controls, while partial remission occurred in 4 of 10 versus 3 of 20, respectively. Overall remission was achieved in all telitacicept-treated patients compared with 45% of controls (*P* = .004).

Hematuria also showed progressive improvement after telitacicept treatment. Individual urinary red blood cell (URBC) trajectories are presented in Fig. 1E. Mean URBC counts decreased from 347.4 ± 531.1/μL at baseline to 55.2 ± 50.0, 63.1 ± 120.0, 22.3 ± 23.9, 11.1 ± 18.5, 4.1 ± 6.1, and 3.0 ± 3.2/μL at months 3, 6, 9, 12, 18, and 24, respectively (Fig. 1F). Significant reductions compared with baseline were observed from month 9 onwards. Telitacicept was well tolerated: one patient reported transient injection-site discomfort, and no severe infection, malignancy, death, or treatment discontinuation occurred.

To assess the robustness of the primary findings, a sensitivity analysis was performed after excluding four patients who received intensive glucocorticoid or immunosuppressive therapy (Fig. 1G). Among the remaining six patients, eGFR showed an overall increase and remained stable during the 24-month follow-up. Compared with baseline, eGFR was significantly higher at all follow-up time points (paired t-test, all *P* < .05).These findings indicate that the improvement in renal function persisted after the potential confounding effects of intensive immunosuppressive treatment, supporting the robustness of the primary analysis. Detailed glucocorticoid and immunosuppressant treatment courses are presented in Fig. S2.

These observations suggest that long-term telitacicept treatment may be associated with sustained reductions in proteinuria and hematuria and more favorable kidney-function trajectories in selected patients with IgAN over 24 months, supporting the biological rationale for targeting BAFF/APRIL-mediated B-cell and plasma-cell activation in this disease [5]. However, the small sample size, retrospective single-center design, and potential residual confounding from concomitant glucocorticoid, immunosuppressive, and supportive therapies preclude firm causal inference. In addition, telitacicept dosing was individualized during follow-up rather than administered according to a uniform regimen, and the cohort did not include patients receiving the currently used 240-mg weekly regimen, limiting the generalizability of the findings across dosing strategies. Longitudinal immunoglobulin measurements were also incomplete because they were not systematically obtained at predefined time points in all patients, restricting comprehensive assessment of long-term immunological safety. Larger prospective controlled studies incorporating standardized treatment protocols, systematic safety monitoring, and longitudinal mechanistic biomarker profiling are therefore warranted to confirm these findings and determine whether telitacicept can modify the long-term progression of IgAN.

## Supplementary Material

sfag309_Supplemental_File
