# Supplementary material for: Long-term kidney outcomes associated with telitacicept in patients with IgA nephropathy: a 24-month retrospective comparative cohort study
Source: Clin Kidney J. 2026 Sep 2;19(10):sfag309. doi: 10.1093/ckj/sfag309 (PMC13627829; doi:10.1093/ckj/sfag309)
Supplement: Supplementary file 1 [file sfag309_supplemental_file.docx]

| Variable | Telitacicept (n=10) | Control (n=20) | P value |
| --- | --- | --- | --- |
| **Demographic characteristics** |  |  |  |
| Age, years | 38.7 ± 10.9 | 43.2 ± 12.8 | 0.325 |
| Female, n (%) | 6 (60%) | 15 (75%) | 0.431 |
| Body mass index, kg/m² | 26.1 ± 4 | 25 ± 4.3 | 0.508 |
| Systolic blood pressure, mmHg | 133.7 ± 23.8 | 134.8 ± 18.2 | 0.900 |
| Diastolic blood pressure, mmHg | 92.8 ± 18.2 | 86 ± 12.5 | 0.303 |
| Hemoglobin, g/L | 125 ± 19.5 | 117.5 ± 17.8 | 0.321 |
| Albumin, g/L | 35.4 ± 3.7 | 35.5 ± 3.7 | 0.918 |
| Serum creatinine, μmol/L | 112 ± 34 | 107.8 ± 39.1 | 0.765 |
| Uric acid, μmol/L | 397.1 ± 79.4 | 373.9 ± 87.6 | 0.474 |
| eGFR, mL/min/1.73 m² | 69.5 (33.4–103.2) | 61.4 (29–126.4) | 1.000 |
| 24-hour urinary protein, g/24 h | 1450 (391–3212) | 1080.5 (330–5572) | 0.965 |
| Urinary red blood cells, /HPF | 148 (7–1711.2) | 89.2 (11.3–1409.4) | 0.630 |
| **Renal pathology (Oxford MEST-C classification)** |  |  |  |
| M1 (Mesangial hypercellularity), n (%) | 10 (100%) | 20 (100%) |  |
| E1 (Endocapillary hypercellularity), n (%) | 3 (30%) | 10 (50%) | 0.440 |
| S1 (Segmental glomerulosclerosis), n (%) | 8 (80%) | 15 (75%) | 1.000 |
| T1/T2 (Tubular atrophy/interstitial fibrosis), n (%) | 6 (60%) | 14 (70%) | 0.690 |
| C (Cellular/fibrocellular crescents), n (%) | 8 (80%) | 15 (75%) | 1.000 |
| **Concomitant medications, n (%)** |  |  |  |
| RAAS inhibitors | 5 (50%) | 7 (35%) | 0.422 |
| SGLT2 inhibitors | 4 (40%) | 9 (45%) | 1.000 |
| Glucocorticoids | 3 (30%) | 11 (55%) | 0.427 |
| Mycophenolate mofetil | 3 (30%) | 11 (55%) | 0.427 |
| Cyclophosphamide | 1 (10%) | 0 (0%) | 0.310 |

Table S1. Baseline characteristics of study participants

Data are presented as mean ± standard deviation, median (range), or n (%). Between-group comparisons were performed using Student's t-test, Mann–Whitney U test, or Fisher's exact test, as appropriate. eGFR, estimated glomerular filtration rate; MEST-C, Oxford classification; RAAS, renin–angiotensin–aldosterone system; SGLT2, sodium-glucose cotransporter 2.

Figure S1 Flowchart of patient selection and propensity score matching in the telitacicept cohort

A total of 1,298 patients with biopsy-confirmed IgA nephropathy between June 2023 and April 2026 were screened, including 126 patients treated with telitacicept and 1,172 patients not treated with telitacicept. After application of the predefined eligibility criteria, 10 telitacicept-treated patients and 394 potential controls were eligible for matching. Propensity score matching was performed at a 1:2 ratio, resulting in 10 patients in the telitacicept group and 20 matched controls for the final analysis.

Figure S2 Individual telitacicept treatment trajectories and concomitant immunosuppressive therapy

Each horizontal bar represents the treatment course of an individual patient from telitacicept initiation. Color-coded segments indicate telitacicept dose and dosing interval over follow-up. Concomitant corticosteroid treatment is shown in purple, mycophenolate mofetil (MMF) in orange, and the diamond denotes a single dose of cyclophosphamide in Case 2. Corticosteroid doses are presented in mg/day, and the total follow-up duration for each patient is shown in the right-hand column. qw, once weekly; q4w, once every 4 weeks; qd, once daily; MMF, mycophenolate mofetil; CTX, cyclophosphamide; d1, day 1.
